# Sensitivity of Soil Respiration to Variability in Soil Moisture and Temperature in a Humid Tropical Forest

**DOI:** 10.1371/journal.pone.0080965

**Published:** 2013-12-02

**Authors:** Tana E. Wood, Matteo Detto, Whendee L. Silver

**Affiliations:** 1 International Institute of Tropical Forestry, USDA Forest Service, Río Piedras, Puerto Rico, United States of America; 2 Fundación Puertorriqueña de Conservación, San Juan, Puerto Rico, United States of America; 3 Smithsonian Tropical Research Institute, Apartado Balboa, Republic of Panama; 4 Department of Environmental Science, Policy and Management, University of California, Berkeley, California, United States of America; Lakehead University, Canada

## Abstract

Precipitation and temperature are important drivers of soil respiration. The role of moisture and temperature are generally explored at seasonal or inter-annual timescales; however, significant variability also occurs on hourly to daily time-scales. We used small (1.54 m^2^), throughfall exclusion shelters to evaluate the role soil moisture and temperature as temporal controls on soil CO_2_ efflux from a humid tropical forest in Puerto Rico. We measured hourly soil CO_2_ efflux, temperature and moisture in control and exclusion plots (n = 6) for 6-months. The variance of each time series was analyzed using orthonormal wavelet transformation and Haar-wavelet coherence. We found strong negative coherence between soil moisture and soil respiration in control plots corresponding to a two-day periodicity. Across all plots, there was a significant parabolic relationship between soil moisture and soil CO_2_ efflux with peak soil respiration occurring at volumetric soil moisture of approximately 0.375 m^3^/m^3^. We additionally found a weak positive coherence between CO_2_ and temperature at longer time-scales and a significant positive relationship between soil temperature and CO_2_ efflux when the analysis was limited to the control plots. The coherence between CO_2_ and both temperature and soil moisture were reduced in exclusion plots. The reduced CO_2_ response to temperature in exclusion plots suggests that the positive effect of temperature on CO_2_ is constrained by soil moisture availability.

## Introduction

In an era of significant and rapid environmental change, understanding biophysical controls on soil respiration is of immense importance. Tropical forests account for approximately one third of the world's soil carbon (C) pool [1], and have the highest soil respiration rates globally [2]. Temperature and soil moisture are known to affect the production and release of carbon dioxide (CO_2_) from tropical forest soils through their effects on soil redox dynamics, diffusion, root and microbial activity as well as C and nutrient availability [3], [4], [5], [6], [7], [8], [9], [10], [11], [12]. While considerable research has addressed seasonal and inter-annual patterns in soil respiration in tropical forests [5], [7], [10], [13], less is known about the role of temperature and precipitation on shorter time-scales (e.g., hours to days) [8], [12].

In the tropics, mean month-to-month temperature variation is generally much smaller than that observed on shorter, diel time-scales (e.g., 2 to 4°C versus 6 to 12°C, respectively) [14]. Kinetic theory suggests that reaction rates increase with increasing temperature [15], [16]. Laboratory incubations of tropical forest soils support this theory, showing increased soil respiration rates with increasing temperature when carbon (C) and nutrients are not limiting [17], [18], [19]. It follows that soil respiration under field conditions will also respond to short-term variation in soil temperature (i.e., hours to days).

Light and temperature tend to co-vary in tropical forest ecosystems. Soil respiration is a combination of root and heterotrophic respiration and thus changes in light availability could drive changes in soil respiration via affects on plant activity. In high latitude ecosystems, light limitation of photosynthesis reduced allocation of photosynthate to roots leading to reduced root respiration [20]. A field study in the eastern Amazon found a weak correlation between soil CO_2_ efflux and temperature on a diel time-scale in an active pasture, but no correlation in neighboring old growth forest or in a degraded pasture [21]. Given the sharp drop in soil CO_2_ efflux that was observed at the end of the daylight period, the authors hypothesized that the diel pattern may be related more to the response of grass metabolism to light than to a response of soil processes to soil temperature. Thus apparent relationships between diel or seasonal variation in soil CO_2_ efflux with temperature may actually be due to effects of light availability on root respiration.

Tropical forests experience a wide range of variation in precipitation, at both short (hour to day) to long (seasonal and interannual) temporal scales. This variability in the timing and magnitude of precipitation events can drive changes in biophysical and biogeochemical conditions that can affect soil CO_2_ effluxes in complex ways [8]. High soil water content creates a barrier at the soil-atmosphere surface, which could inhibit the diffusion of CO_2_ out of the soil [8], [9], [22]. In humid tropical forests, the consistently moist conditions combined with finely textured clay soils and high biological demand for oxygen (O_2_) can facilitate the periodic depletion of O_2_ in surface soils [23], [24]. Declines in soil O_2_ concentrations have been found to occur within hours of even small precipitation events (∼1 mm) [24]. Low soil O_2_ availability can limit aerobic respiration, decreasing soil CO_2_ effluxes [25]. However, highly weathered tropical forests are typically rich in poorly crystalline, reactive iron (Fe) minerals; declines in soil redox potential in humid tropical forest soils can drive high rates of iron (Fe) reduction and anaerobic CO_2_ respiration [26]. In controlled laboratory experiments, rates of CO_2_ production under anaerobic conditions were similar to rates of aerobic respiration [27]. Iron reduction can also increase soil phosphorus (P) availability by decreasing the affinity of Fe for P. Biological activity is generally assumed to be limited by P in these ecosystems [28], [29], and thus alleviation of P limitation during low or fluctuating redox conditions has the potential to fuel increased soil respiration [30], [31], [32], [33].

Moisture limitation can also reduce microbial activity and restrict microbial access to C substrates [34], [35]. The associated increase in O_2_ diffusion into dry soils would increase the concentration of oxidized Fe, decrease P availability through Fe-P bonding, and potentially limit CO_2_ production [30], [33]. Although the relationships between moisture and soil respiration are complex, theory generally predicts a parabolic relationship between soil CO_2_ efflux and soil moisture with the highest soil CO_2_ emissions occurring at an intermediate moisture level [7], [36], [37], [38], [39]. During periods of soil water saturation and extreme soil drying soil moisture is likely to exert a stronger control over soil CO_2_ efflux than that of soil temperature [10].

In this study we investigated hourly to daily changes in soil CO_2_ efflux in a relatively a-seasonal humid tropical forest in Puerto Rico to determine (1) the timescale over which CO_2_ efflux varies, (2) the relationship of this variation to soil temperature and moisture, and (3) how these relationships are affected by experimental reduction in soil moisture.

## Materials and Methods

### Site Description

We conducted this research in the Bisley Experimental Watersheds of the Luquillo Experimental Forest in northeastern Puerto Rico (18°18′N, 65°50′W) [33]. Permission to work in this site was granted by the USDA Forest Service International Institute of Tropical Forestry. This research was conducted in collaboration with the Luquillo Long-term Ecological Research (Luq-LTER), and as such, data will be made available via the Luq-LTER database (http://luq.lternet.edu/data/datacatalog). The forest is classified as subtropical wet forest [40]. The elevation is approximately 300 m above sea level, receives an average 3500 mm of precipitation annually, and the mean annual temperature is 23°C. Mean month-to-month variation in temperature is approximately 4°C throughout the year. While precipitation is highly variable throughout the year, there is no significant dry season [41]. The soils are deep, highly weathered, clay-rich and acidic. The study site was dominated by the palm *Prestoea montana* R. Graham Nichols [33].

### Experimental Design

We created an experimental drought using small (1.54 m^2^) throughfall exclusion shelters that were in place from June through August of 2008 (3 months total). This study included a total of three exclusion and three control plots. We used time-domain reflectometery (TDR, Campbell Scientific Model CS616) to estimate hourly soil moisture in all plots (0–30 cm), and measured hourly soil temperature (10 cm; Campbell Scientific, Model 108 L) in one control and one exclusion plot. Automated soil respiration chambers (Li-Cor LI-8100/8150 Multiplexer; Li-Cor Biosciences, Lincoln, NE, USA) were installed in all six plots to measure hourly changes in soil CO_2_ efflux. Due to limited power access at the field site, soil CO_2_ efflux was measured in a series of field campaigns conducted over a six-month period that included three months with the throughfall exclusion shelters in place and three months following shelter removal (average 8 days per campaign, 110 days total). For a more detailed description of the study site and methodology, see Wood and Silver [33].

### Statistical Analyses

The variance of each time series (e.g, temperature, moisture, respiration) was decomposed on a scale-by-scale basis using orthonormal wavelet transformation (Matlab version 7.0 [R2010a], Mathworks; Appendix S1). This spectral technique, analogous to Fourier analysis, breaks the process variance into pieces, each of which represents the contribution on a particular scale [42]. Given a time series *X_t_* (*t = 0,2,* …,*N−1*) that is regarded as a stochastic process with stationary increment, and a unit level Daubechies wavelet filter *h_1,l_* of width *L*, the wavelet variance at the *j-*scale *τ_j_ = 2^j−1^* is defined as: 
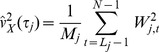
(1)where *M_j_ = N−L_j_+1 *and *L_j_ = (2^j^−1)(L−1)−1.*

The coefficients *W* are computed as:
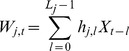
(2)


**Figure 1:**
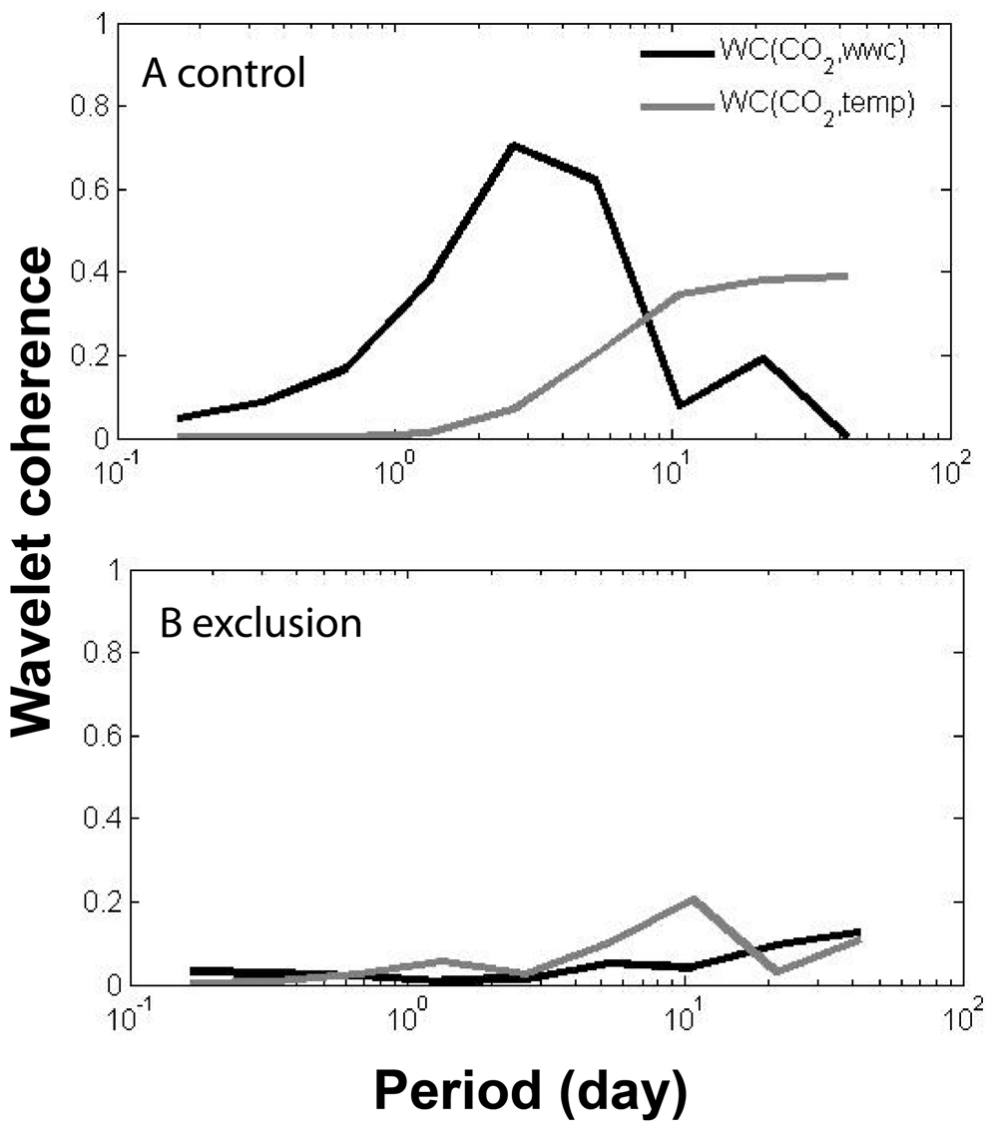
The wavelet coherence [44] between CO_2_ efflux and soil moisture (black) and temperature (gray) in the (A) control and (B) exclusion plots.

We used the Mondal and Percival [43] method to compute an unbiased estimator of the wavelet variance for gappy series where the missing values are replaced by zeros (48% of data; Fig. 1):
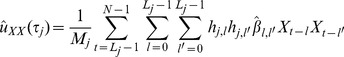
(3)where 
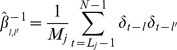
 and 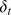
assumes the value zero or unity, with zero indicating that 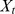 is missing. In the bi-variate case the wavelet co-variance between two time series *X* and *Y* is defined as:
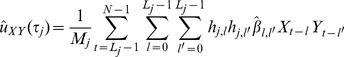
(4)


A normalized wavelet covariance (the analogy of the coefficient of correlation) can be obtained combining equation (3) and (4) to form [44]:
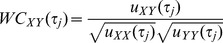
(5)


The correlation among variables are explored in the following using the Haar-wavelet coherence defined in equation (5). We used regression analyses to determine relationships between CO_2_ efflux (mean of three replicates per treatment) and mean soil characteristics (e.g., soil moisture and temperature). When significant diel variation was observed, regressions were performed using mean hourly values, all other regressions performed using mean daily values. All regressions were performed using SigmaPlot 10 (SigmaPlot for Windows, v. 7.101, 2001, SPSS Inc.).

## Results

**Figure 2:**
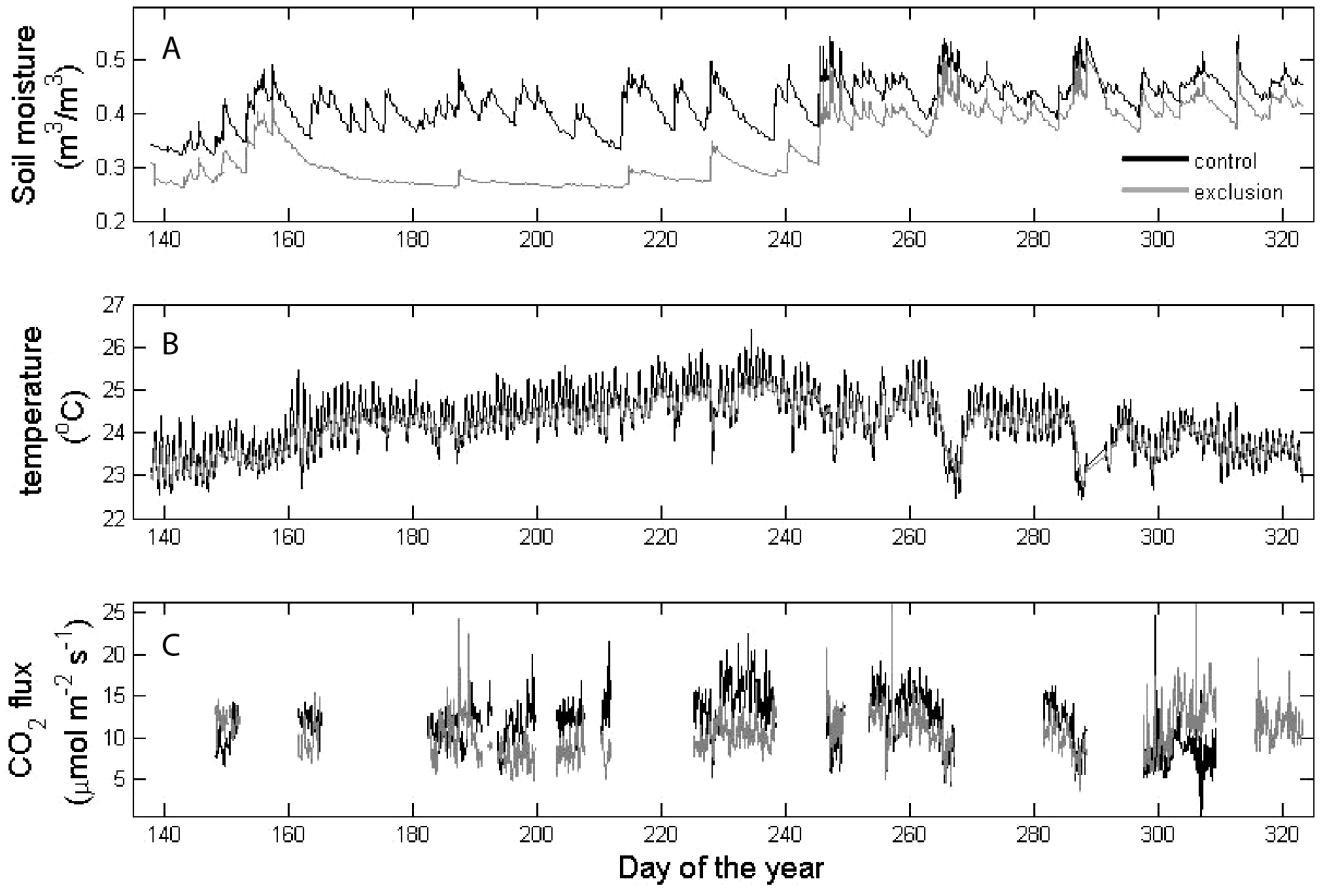
Time series for (A) soil moisture, (B) soil temperature, and (C) carbon dioxide (CO_2_) flux over the 6-month study (June through December 2008) in the control (black) and exclusion (gray) plots. Throughfall exclusion shelters were in place from June through August (3-months).

**Figure 3:**
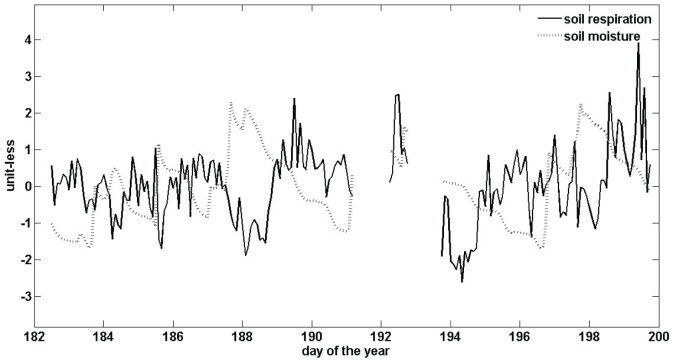
Plot of soil moisture and soil respiration for one of the eight field campaigns (18-days, June 30-July 18, 2008). Soil respiration declined following large rainfall events and the subsequent increase in soil moisture.

We found no significant diel periodicity in soil respiration. Soil CO_2_ efflux did, however, display significant periodicity over daily to seasonal time-scales. Soil respiration in control plots showed high coherence with soil moisture for a broad range of time scales, with a peak correlation corresponding to a two-day periodicity (Fig. 1). This two-day periodicity is the timescale over which strong fluctuations in volumetric soil moisture occurred (Fig. 2A). Further analyses of the time series revealed a negative relationship between soil moisture and soil CO_2_ efflux (Fig. 3). Soil respiration and temperature were correlated on a scale of weeks to months, with peak respiration occurring during the period of highest temperatures (Fig. 2).

**Figure 4:**
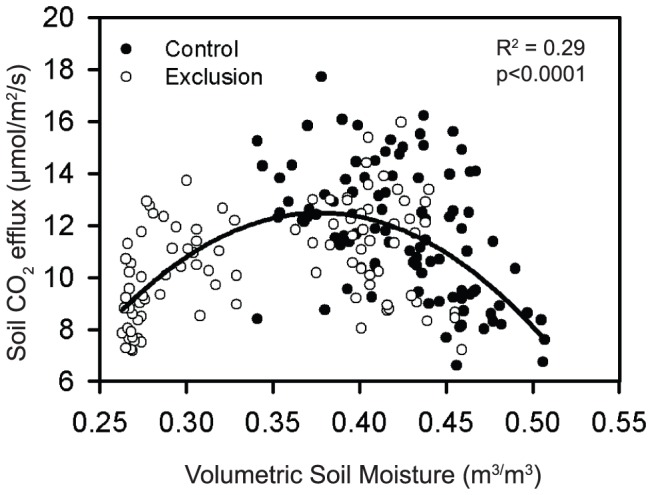
Regression between mean daily volumetric soil moisture and mean daily soil CO_2_ efflux in both the control (solid circles) and exclusion (open circles) plots. The equation for the regression is f = y_0_+a_*_x+b_*_x^2^.

**Figure 5:**
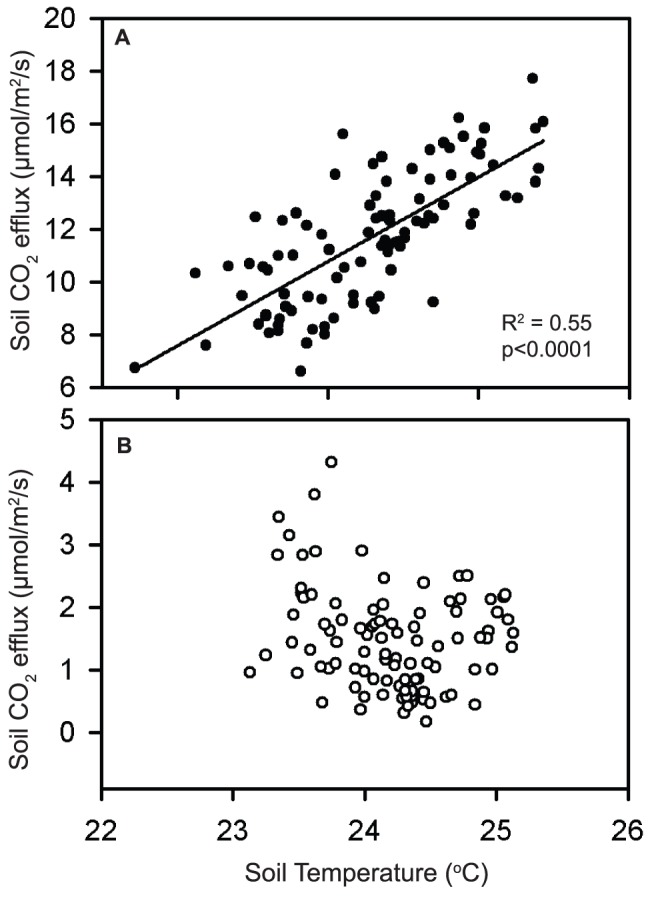
Regression between mean daily soil temperature and mean daily soil CO_2_ efflux in the (A) control and (B) exclusion plots.

Throughfall exclusion reduced volumetric soil moisture by an average of 29% relative to the controls. There was reduced coherence between soil respiration and soil temperature, as well as with soil moisture in these plots. There was a significant parabolic relationship between mean daily volumetric soil moisture and mean daily soil CO_2_ efflux when both treatments were included, with peak soil respiration occurring when volumetric soil moisture was approximately 0.375 m^3^/m^3^ (Fig. 4, R^2^ = 0.29, p<0.0001, f = y_0_+a_*_x+b_*_x^2^). Variation in the residuals was significantly and positively related to soil temperature (R^2^ = 0.15, P<0.0001). The same relationship between soil moisture and soil CO_2_ efflux was found when the control and exclusion plots were evaluated separately (R^2^ = 0.29, p<0.0001 [control]; R^2^ = 0.28, p<0.0001 [exclusion]). We found a significant, positive linear relationship between mean daily soil temperature and mean daily CO_2_ efflux in the control plots, but not in the exclusion plots (R^2^ = 0.55, p<0.0001, Fig. 5).

## Discussion

We found no significant diel variation in soil respiration in this forest. This is in contrast to findings in temperate and boreal forests, which have found that soil respiration varied with soil temperature and photosynthesis on diel time-scales [8], [45], [46], [47], [48], [49]. Significant diel variation has also been found in some tropical forest sites [8], [50], [51], but not all (this study, [9], [21]). The lack of a consistent diel response of soil CO_2_ efflux across tropical forest sites could be due to differences in the magnitude of the diel change in temperature across these forest sites, variability in the relative contribution of root versus microbial respiration to total soil respiration, or because other factors such as soil moisture status, exert a stronger control over total soil respiration in some systems than temperature. Furthermore, root and soil respiration have been found to demonstrate differential responses to environmental variables (e.g., soil moisture) [52], [53], [54]. Hence, while we observed no significant diel pattern of net soil respiration, partitioning soil respiration into its components (e.g., litter, root and soil) could reveal different results [53].

We found significant coherence between soil CO_2_ efflux and soil moisture on a two-day time-scale. The periodicity of this relationship corresponds with large rainfall events that significantly increased volumetric soil moisture and lowered soil CO_2_ efflux (e.g., Fig. 3). Rapid declines in soil CO_2_ efflux in response to soil water saturation has been observed in seasonal forest in the Amazon [8] and in moist tropical forest in Panama [9]. The decline in soil CO_2_ efflux in response to increased volumetric soil moisture could be the result of reduced diffusion of CO_2_ from saturated soils [8], [9], [10]. Reduced soil CO_2_ efflux could also be due to reduced soil microbial activity in low O_2_ environments [25].

As expected, we found a significant parabolic relationship between soil moisture and soil CO_2_ efflux with peak soil respiration occurring when volumetric soil moisture was at an intermediate value of approximately 0.375 m^3^/m^3^ (Fig. 5). This parabolic relationship between soil moisture and soil CO_2_ efflux agrees with findings from other tropical forest sites [6], [8], [36]. Interestingly, the “tipping point” of the positive effect of soil moisture on CO_2_ efflux is similar across tropical forests on clay soils, occurring at mean volumetric soil moisture values of approximately 0.35 m^3^/m^3^ (this study) [7], [36] to 0.45 m^3^/m^3^ [6], [8]. In many soils, when the soil moisture content is at about 40%, a small increase in soil moisture content leads to a large increase in soil resistance to the diffusion of gases, thereby reducing soil CO_2_ emissions [9], [12], [55]. When tropical forests on sandy soils are considered, this tipping point is reduced (0.22 m^3^/m^3^) [7]. These findings would suggest that soil texture plays an important role in determining the tipping point of the positive effect of soil moisture on soil CO_2_ efflux in tropical soils.

The observed trend of a positive coherence between soil respiration and temperature on seasonal timescales is intriguing given the low variability in temperature during the study period (Fig. 1; 2°C) [33]. Despite this low seasonal variability, there is evidence that soil respiration in tropical forest sites exhibits a positive response to relatively small increases in temperature over monthly to annual time-scales [6], [7], [8], [10]. However, temperature and light tend to co-vary in tropical forests (Silver et al. *unpublished data*) [7], hence it is also possible that the observed relationship between temperature and soil respiration is driven by variation in light via the positive effect of light on photosynthesis and the resulting increase in carbohydrate allocation to roots rather than temperature. However, experimental manipulation of temperature in a field setting would be needed to distinguish the effects of temperature versus those of light availability on soil CO_2_ efflux in tropical systems. Currently, no field-warming experiment has been conducted in a tropical forest [18], [56], [57].

Interestingly, the coherence between soil moisture and soil respiration on a two-day time-scale was reduced significantly in the exclusion plots (Fig. 1). This reduced coherence could be due to the filtering out of the effects of large rainfall events on soil moisture availability in the exclusion plots (Fig. 2; Days 160 to 240). This finding would suggest that considering the temporal variability of precipitation events in addition to the role of total precipitation inputs is important when evaluating moisture controls on soil CO_2_ efflux. In addition to a reduced coherence between soil moisture and CO_2_ efflux, there was also a reduction in the CO_2_ response to temperature in the exclusion plots, which suggests that the positive effect of temperature on CO_2_ efflux at weekly to monthly time scales is constrained by soil moisture. This result is supported by the significant positive relationship between soil temperature and soil CO_2_ efflux in the control plots, but not the exclusions (Fig. 5). Interestingly, when we evaluated soil respiration on a weekly time-scale, we found no significant influence of soil moisture or temperature on soil respiration and no significant differences in soil respiration between the control and exclusion plots [33], which highlights the value of collecting soil CO_2_ efflux measurements with high temporal resolution. Overall, the reduced coherence of soil moisture and soil temperature with soil CO_2_ efflux in the exclusion plots suggest that small reductions in soil moisture availability can result in moisture availability as a predominant limiting factor of soil CO_2_ efflux in tropical soils, even in sites that receive relatively large rainfall inputs throughout the year.

## Conclusions

Overall, higher soil moisture led to lower soil CO_2_ emissions in this study. The reduction in CO_2_ release could be the result of abiotic and biotic factors. The reduced water-filled pore space of the saturated soil may have decreased diffusion of CO_2_ out of the soil, leading to lower CO_2_ emissions [9], [24]. Saturated soils also limit the diffusion of O_2_ into the soil, which could have created anaerobic conditions that limit the production of CO_2_ [23], [25]. Continued dry down of soils has been shown to reduce CO_2_ emissions from some tropical forests, but has had no effect in others [3], [33], [52], [54], [58]. Our results highlight the strong sensitivity of soil respiration to short-term dynamics in soil moisture and longer-term patterns in temperature or light availability in a humid tropical forest. Our results also show that the well-established relationship of soil respiration to temperature is changed when soil moisture is reduced. This finding would suggest that temperature exerts a positive control on soil respiration as long as soil moisture is not limiting. Determining which processes will dominate in tropical forests depends heavily on our ability to accurately predict how climate change will affect precipitation patterns and hydrologic cycles in these ecosystems.

## Supporting Information

Appendix S1 **Matlab code for presented analyses.**
(DOC)
