# Supplementary material for: Sensitivity of Soil Respiration to Variability in Soil Moisture and Temperature in a Humid Tropical Forest
Source: PLoS One. 2013 Dec 2;8(12):e80965. doi: 10.1371/journal.pone.0080965 (PMC3846571; doi:10.1371/journal.pone.0080965)
Supplement: Appendix S1 — Matlab code for presented analyses. (DOC) [file pone.0080965.s001.doc]

Appendix A: Matlab code for presented analyses.

% compute Haar wavelet spectrum for gappy series

%

% based on Mondal and Pervival (2010)

% Wavelet Variance Analysis for Gappy Time Series

%

% written by: Matteo Detto PhD

% Biometeorology lab, Ecosystem Science Division

% Dept. of Environmental Science, Policy, and Management (ESPM)

% University of California, Berkeley, CA 94720

% Phone: 1-510-642 9048

% Fax: 1-510-643-5098

% last update: 09 Oct 2011

function [n2Xg,tau] = HAAR_spec_gappy_v2(X1,varargin)

%default options

opts=struct('L',2,...          Deabouchies filter (with L=2, Haar)

           'dt',1,...         data interval

           'J1',-1,...        %max scale default floor(log2(N))

           's0',1);           %min scale

opts=parseArgs(varargin,opts);

tic

%constant and parameters

X(:,1)=X1-nanmean(X1);

N=length(X);

delta=ones(N,1);

delta(isnan(X))=0.0;

X(isnan(X))=0.0;

if opts.J1==-1

   J=min(floor(log2(N)),9);

else

   J=opts.J1;

end

dt=opts.dt;

s0=opts.s0;

L=2;

% h1=MakeWavelet(0,0,'Haar',0,'Mother',L);

n2Xg=nan(1,J-s0+1);

tau=zeros(1,J-s0+1);

if sum(delta)<N

%setup loop arrays

Lj=(2^J-1)*(L-1)+1;

%all combinations of l and lp

A = allcomb(1:Lj,1:Lj);

l  = A(:,1);

lp = A(:,2);

use=find(l>=lp);

l  = l(use);

lp = lp(use);

%weight function 2 for l>lp and 1for l==lp

w=ones(length(l),1)*2;

w(l==lp)=1;

 wb = waitbar(0,'constructing beta function...');

 beta_1=ones(Lj,1);

%calculate beta array fo l and lp loops

for k = 1:Lj

    waitbar(k/length(l))

   %beta(k,1)=Mj/(delta(Lj-l(k)+1:N-l(k)+1)'*delta(Lj-lp(k)+1:N-lp(k)+1));

    d1=delta(1:N-k+1)';

    d2=delta(k:N);

    beta_1(k,1)=d1*d2/(N-k+1);

end

close(wb)

for j2=s0:J

 j=J-j2+s0;

   tau(j)=2^j*dt;

   Lj=(2^j-1)*(L-1)+1;

   lj=[0:Lj-1]';

   Mj=N-Lj+1;

   jmax=ceil(log2(Lj));

%     h=MakeWavelet(0,0,'Haar',L,'Mother',2^jmax);

%  MakeWavelet Haar

   h=ones(1,2^jmax)/sqrt(2^jmax);h(1:2^jmax/2)=-h(1:2^jmax/2);

   %setup loop arrays

   t = Lj:N;

   use=find(l<=Lj & lp<=Lj & beta_1(l-lp+1)~=0);

   if isempty(use)==0

       l  = l(use);

       lp = lp(use);

       w  = w(use);

       %setup hj,l and hj,lp arrays - do not change with t

       clear hl hlp beta

       hl(:,1)  = h(l);

       hlp(:,1) = h(lp);

       beta(:,1)= 1./beta_1(l-lp+1);

       %clear output

       n3Xg=0.0;

       wb = waitbar(0,['internal loop j:' num2str(j)]);

       %loop through t

       for i = 1:length(t)

          waitbar(i/length(t));

           %setup t-l and t-lp arrays

           tml = t(i) -l  + 1;

           tmlp =t(i) -lp + 1;

        use=find(X(tml)~=0 & X(tmlp)~=0);

        if isempty(use)==0

        %calculate Wp array for l and lp loops and add sum to output

%        Wp = (hl.*hlp.*X(tml).*X(tmlp).*beta.*delta(tml).*delta(tmlp))/Mj;

        %Wp = (hl(use).*hlp(use).*X(tml(use)).*X(tmlp(use)).*beta(use).*w(use));

        Wp = (hl(use).*hlp(use).*(X(tml(use))-X(tmlp(use))).^2.*beta(use).*w(use));

        n3Xg=n3Xg+sum(Wp);

        end

  %  n2Xg(j)=n3Xg/Mj;

    n2Xg(j)=-n3Xg/Mj/2;

   end

         close(wb)

   end

end

toc

else

   for j=1:J

       J-j

   tau(j)=2^j*dt;

   Lj=(2^j-1)*(L-1)+1;

   lj=[0:Lj-1]';

   Mj=N-Lj+1;

   jmax=ceil(log2(Lj));

%     h=MakeWavelet(0,0,'Haar',L,'Mother',2^jmax)

%  MakeWavelet Haar

   h=ones(1,2^jmax)/sqrt(2^jmax);h(1:2^jmax/2)=-h(1:2^jmax/2);

     W=zeros(N-Lj+1,1);

    for t=Lj:1:N

      W(t-Lj+1)=h*X(t-Lj+1:t);

    end

      n2Xg(j)=mean(W.^2);

   end

end
